# Young Woman With Shortness of Breath

**DOI:** 10.1016/j.acepjo.2026.100516

**Published:** 2026-10-02

**Authors:** Abrianna Hite, J. David Gatz

**Affiliations:** 1 Ohio Wesleyan University, Delaware, Ohio, USA; 2 Department of Emergency Medicine, University of Maryland School of Medicine, Baltimore, Maryland, USA

## 1 Case Presentation

A 22-year-old woman presented to the emergency department with sudden onset of shortness of breath that began 12 hours before presentation, associated with positional chest pain. She denied any trauma or infectious symptoms. Her only significant past medical history included severe dysmenorrhea and vitamin D deficiency. There was no history of smoking or asthma. Physical exam was notable for tachypnea and tachycardia, and an absence of wheezing on auscultation. An urgent chest radiograph showed bilateral abnormalities in the lungs (Fig).

**Figure:**
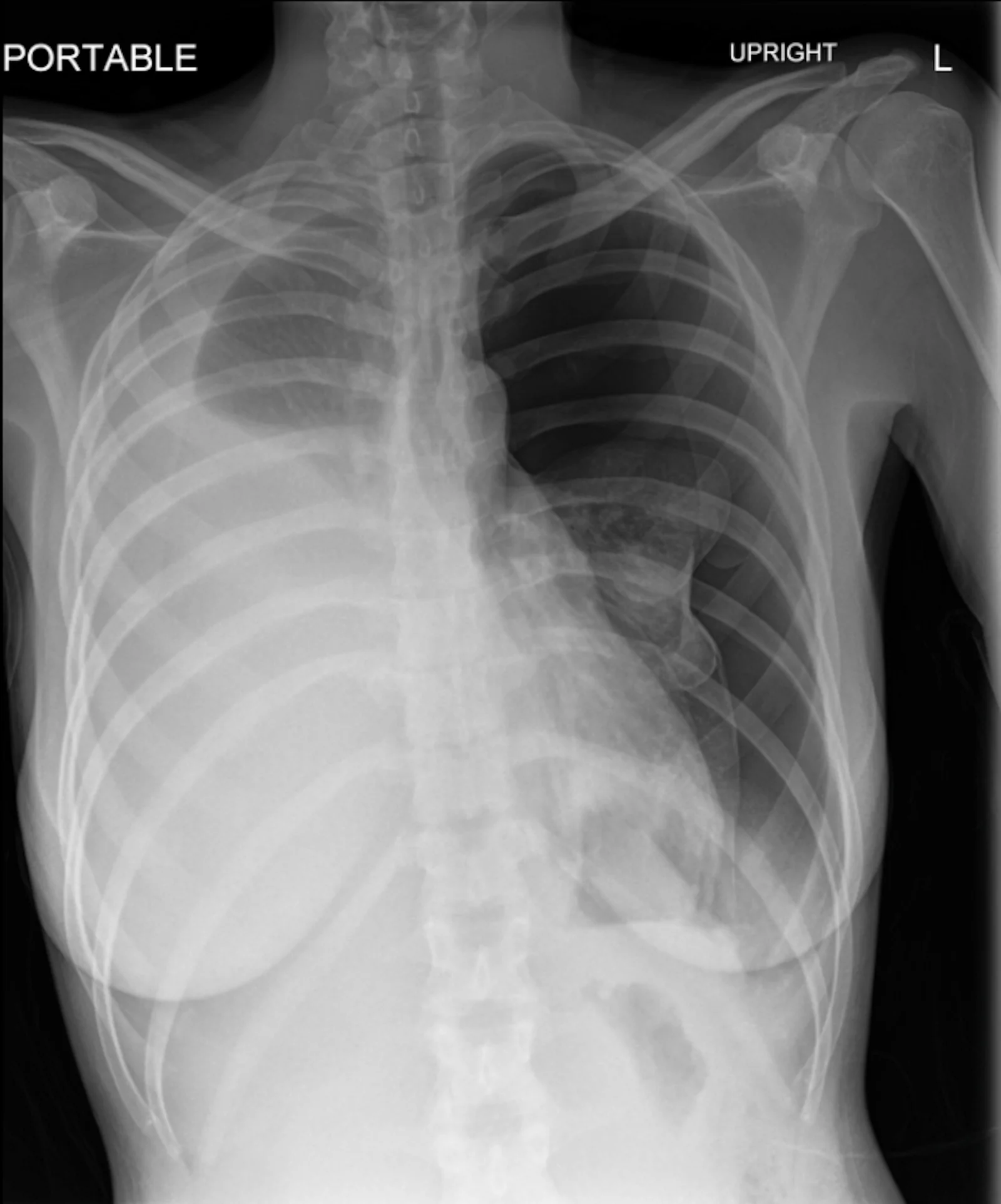
Chest radiograph with abnormalities to the bilateral lungs showing catamenial pneumothorax.

## 2 Diagnosis: Thoracic Endometriosis Syndrome (TES) With Simultaneous Catamenial Pneumothorax And Contralateral Catamenial Hemothorax

Catamenial pneumothorax and hemothorax are rare manifestations of TES.^1^^,^^2^ Of the two, catamenial pneumothorax is more common, and characteristically associated with the window immediately before and days following onset of menses. Notably, there is a profound right-sided predisposition, with approximately 90% of manifestations occurring in the right hemithorax.^3^ The simultaneous development of catamenial pneumothorax and contralateral hemothorax is exceedingly rare, with only one prior case identified in the literature.^4^

There are multiple theories as to how endometrial tissue reaches the thorax, and similarly multiple theories regarding the development of catamenial pneumothoraces. Catamenial hemothorax, by contrast, is almost always associated with functional endometrial tissue within the thoracic cavity.^3^ Diagnosis in the emergency department (ED) setting may be delayed due to nonspecific symptoms or mistaking a presentation for a primary spontaneous pneumothorax. Clinicians should maintain a high index of suspicion in women of reproductive age, particularly when symptoms demonstrate a temporal relationship to the menstrual cycle. The need for immediate intervention in the ED will depend on patient stability, but long-term management usually requires a multidisciplinary team and may include surgical interventions (eg, video-assisted thoracoscopic surgery, hysterectomy, and bilateral salpingo-oophorectomy) and pharmacologic/hormonal therapies.

## Funding and Support

By *JACEP Open* policy, all authors are required to disclose any and all commercial, financial, and other relationships in any way related to the subject of this article as per ICMJE conflict of interest guidelines (see www.icmje.org). The authors have stated that no such relationships exist.

## Conflict of Interest

All authors have affirmed they have no conflicts of interest to declare.
